# The Bif-1-Dynamin 2 membrane fission machinery regulates Atg9-containing vesicle generation at the Rab11-positive reservoirs

**DOI:** 10.18632/oncotarget.8028

**Published:** 2016-03-10

**Authors:** Yoshinori Takahashi, Nikolaos Tsotakos, Ying Liu, Megan M. Young, Jacob Serfass, Zhenyuan Tang, Thomas Abraham, Hong-Gang Wang

**Affiliations:** ^1^ Department of Pediatrics, Penn State University College of Medicine, Hershey, PA 17033, USA; ^2^ Department of Pharmacology, Penn State University College of Medicine, Hershey, PA 17033, USA; ^3^ Department of Neural and Behavioral Science and the Microscopy Imaging Facility, Penn State University College of Medicine, Hershey, PA 17033, USA; ^4^ Penn State Cancer Institute, Penn State University College of Medicine, Hershey, PA 17033, USA

**Keywords:** autophagy, Atg9 vesicle, Bif-1, Dynamin 2, Rab11-positive reservoir

## Abstract

Atg9 is a multispanning transmembrane protein that is required for autophagosome formation. During autophagy, vesicles containing Atg9 are generated through an unknown mechanism and delivered to the autophagosome formation sites. We have previously reported that Atg9-containing membranes undergo continuous tubulation and fission during nutrient starvation in a manner dependent on the curvature-inducing protein Bif-1/Sh3glb1. Here, we identify Dynamin 2 (DNM2) as a Bif-1-interacting protein that mediates the fission of Atg9-containing membranes during autophagy. The interaction of Bif-1 and DNM2 is enhanced upon nutrient starvation, and Bif-1 and DNM2 cooperatively induce the generation of Atg9-containing vesicles. Inhibition of the GTPase activity of DNM2 results in the accumulation of Atg9-positive tubular structures that originate from a Rab11-positive reservoir. Although Atg9 seems to be constitutively trafficked to the reservoir regardless of Bif-1 expression, membrane tubulation from the Atg9 reservoir is dependent on Bif-1 and is strongly induced upon nutrient starvation. These findings suggest that the generation of Atg9 vesicles from a Rab11-positive reservoir is tightly controlled by the Bif-1-DNM2 membrane fission machinery in response to cellular demand for autophagy.

## INTRODUCTION

Autophagy is an evolutionarily conserved lysosomal catabolic pathway essential for the maintenance of cellular and tissue homeostasis [1]. Autophagy is initiated in response to a variety of intracellular and extracellular stresses including nutrient starvation. The process of autophagy begins with the sequestration of cytoplasmic components into a crescent-shaped, double-membrane structure termed the phagophore. The phagophore is extended and sealed to form a double-membrane vesicle called the autophagosome, which ultimately fuses with a lysosome for hydrolytic degradation of the sequestered materials. The resulting macromolecules are subsequently transported back into the cytoplasm for reuse [2].

The mechanism of autophagosome biogenesis, specifically the origin and expansion of the autophagosomal membrane, has been a hot topic in the field [3]. Recently, Atg9-containing vesicles (Atg9 vesicles) produced de novo during starvation have been shown to contribute to the formation of autophagosomes [3, 4]. Atg9 is an N-Glycosylated membrane protein that is thought to deliver lipids (and possibly regulators) required for the elongation and sealing of phagophores [3, 5]. In yeast, Atg9 must traffic through and exit the Golgi in order to exert its function in autophagy [6]. Moreover, the post-Golgi pool of yeast Atg9 has been found to localize on novel tubular-vesicular organelles referred to as the Atg9 reservoir [7]. In mammalian cells, Atg9 mainly resides in the juxtanuclear Golgi region but is trafficked to the peripheral cytoplasmic region in response to nutrient starvation [8]. Although the mechanism responsible for Atg9 trafficking remains unclear, Atg9 vesicles in the cytoplasmic region have been found to colocalize with the phagophore and autophagosome markers Atg16L1 and LC3 [9]. Interestingly, recent studies have suggested that mammalian Atg9 is also present on a unique recycling endosome-like tubular vesicular compartment that has similar properties as the yeast Atg9 reservoirs [9–11].

The amino-terminal bin-amphiphysin-rvs (N-BAR) protein family, which forms a crescent-shape structure capable of deforming lipid bilayers, has emerged as a key regulator of membrane fission events [12]. Bif-1 (also known as Sh3glb1/Endophilin B1) is a member of Endophilin protein family that contains N-BAR and Src homology 3 (SH3) domains. The SH3 domain is a well-characterized motif to mediate protein-protein interactions [13]. Recently, we reported that Bif-1 is a critical regulator of Atg9 vesicle formation during autophagy [9]. We observed that the juxtanuclear Atg9-containing compartments undergo Bif-1-dependent budding upon nutrient starvation to form Atg9 vesicles. Bif-1 mutants deficient in membrane binding or in the curvature-inducing region of the N-BAR domain fail to restore starvation-induced Atg9 vesicle formation in *Bif-1*-depleted cells, suggesting the importance of Bif-1-mediated membrane budding for Atg9 trafficking. Interestingly, our previous data also show that the N-BAR domain of Bif-1 is, itself, not sufficient for nutrient starvation-induced Atg9 trafficking; therefore, protein complex formation through the SH3 domain of Bif-1 may be a critical step in regulating Atg9 vesicle formation during autophagy.

Here, we identified Dynamin 2 (DNM2) as a Bif-1-interacting protein and provide evidence that the Bif-1-DNM2-mediated membrane fission machinery plays a critical role in generating Atg9 vesicles during autophagy. We found that the Rab11-positive compartment serves as the reservoir for the Bif-1-DNM2-dependent generation of Atg9 vesicles required for proper autophagic flux.

## RESULTS

### Bif-1 interacts with DNM2 via its SH3 domain

**Figure 1:**
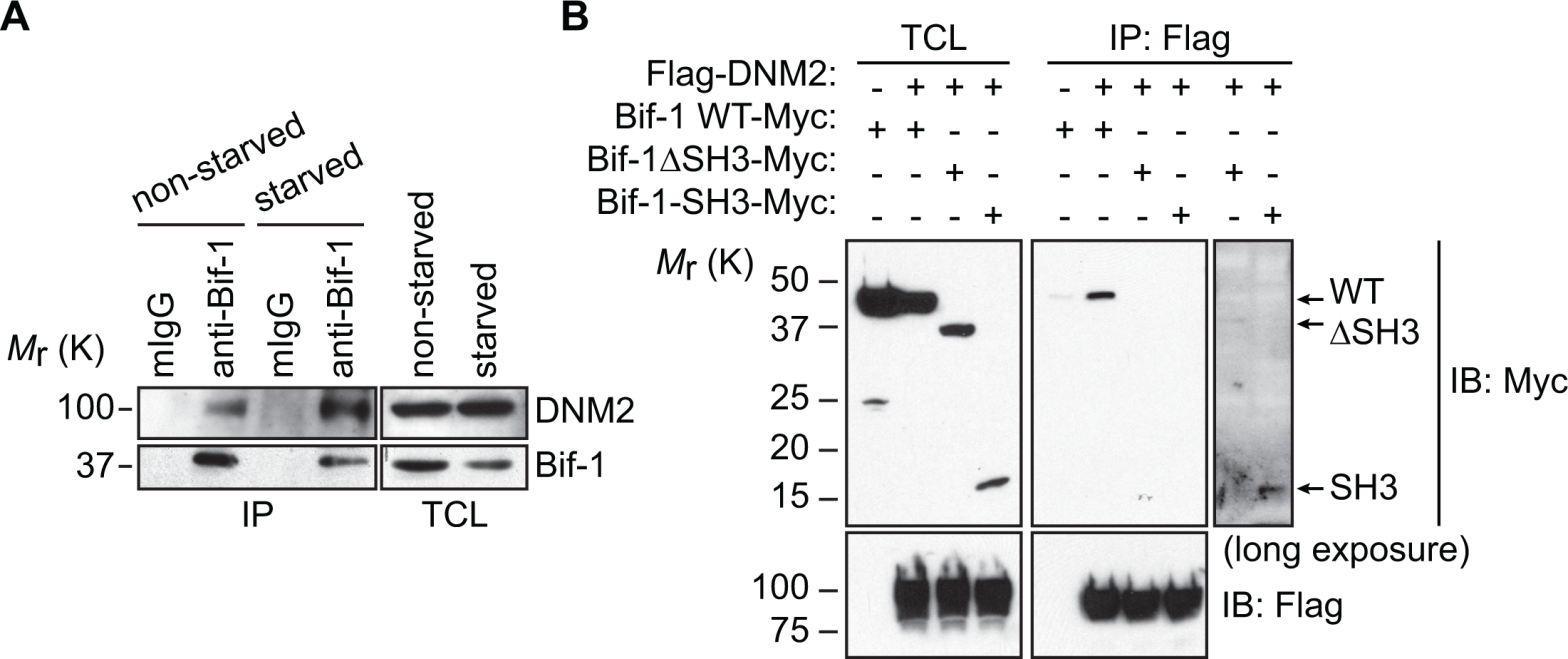
Identification of DNM2 as a Bif-1-interacting protein (**A**) HeLa/S cells were incubated in starvation medium (starved) or control complete medium (non-starved) for 2 h and subjected to co-immunoprecipitation using anti-Bif-1 antibodies or control mouse IgG followed by immunoblotting using the indicated antibodies. (**B**) 293T cells were transiently co-transfected with the indicated plasmids and subjected to co-immunoprecipitation using anti-Flag antibodies followed by immunoblotting using the indicated antibodies.

To understand the molecular mechanism of Atg9 vesicle generation, we performed tandem affinity purification using TAP-tagged (CBP (calmodulin binding peptide)-TEV-IgG-binding domain) Bif-1 fusion protein as bait. Mass spectrometry analysis identified several vesicle trafficking-related proteins as putative Bif-1-interacting proteins including a large GTPase, Dynamin (DNM) 2. Since DNMs are known to cooperate with N-BAR containing proteins during membrane fission [12], we decided to explore the potential role of DNM2 in Bif-1-mediated autophagy. We first confirmed the endogenous interaction of Bif-1 with DNM2 by co-immunoprecipitation using a Bif-1-specific antibody (Figure 1A). While nutrient (amino acids and serum) starvation decreased the protein expression of Bif-1, the amount of Bif-1 co-precipitated with DNM2 was increased, indicating an enhanced complex formation of DNM2 and Bif-1 during autophagy.

DNM2 contains a proline-rich domain known to interact with SH3 domain-containing proteins [12]. To determine if the SH3 domain of Bif-1 is responsible for interacting with DNM2, 293T cells were co-transfected with Flag-DNM2 and Bif-1 or Bif-1 deletion mutants and subjected to co-immunoprecipitation using an anti-Flag antibody. As shown in Figure 1B, loss of the SH3 domain of Bif-1 (ΔSH3) abrogated the interaction between Bif-1 and DNM2. Moreover, despite having a greater expression of Bif-1 (ΔSH3)-Myc compared to Bif-1-SH3-Myc likely due to protein instability, DNM2 was observed to preferentially interact with Bif-1-SH3-Myc upon longer exposure of the immunoblot, suggesting that the SH3 domain is important for the interaction of Bif-1 and DNM2.

### DNM2 is required for the generation of Atg9 vesicles during nutrient starvation

**Figure 2:**
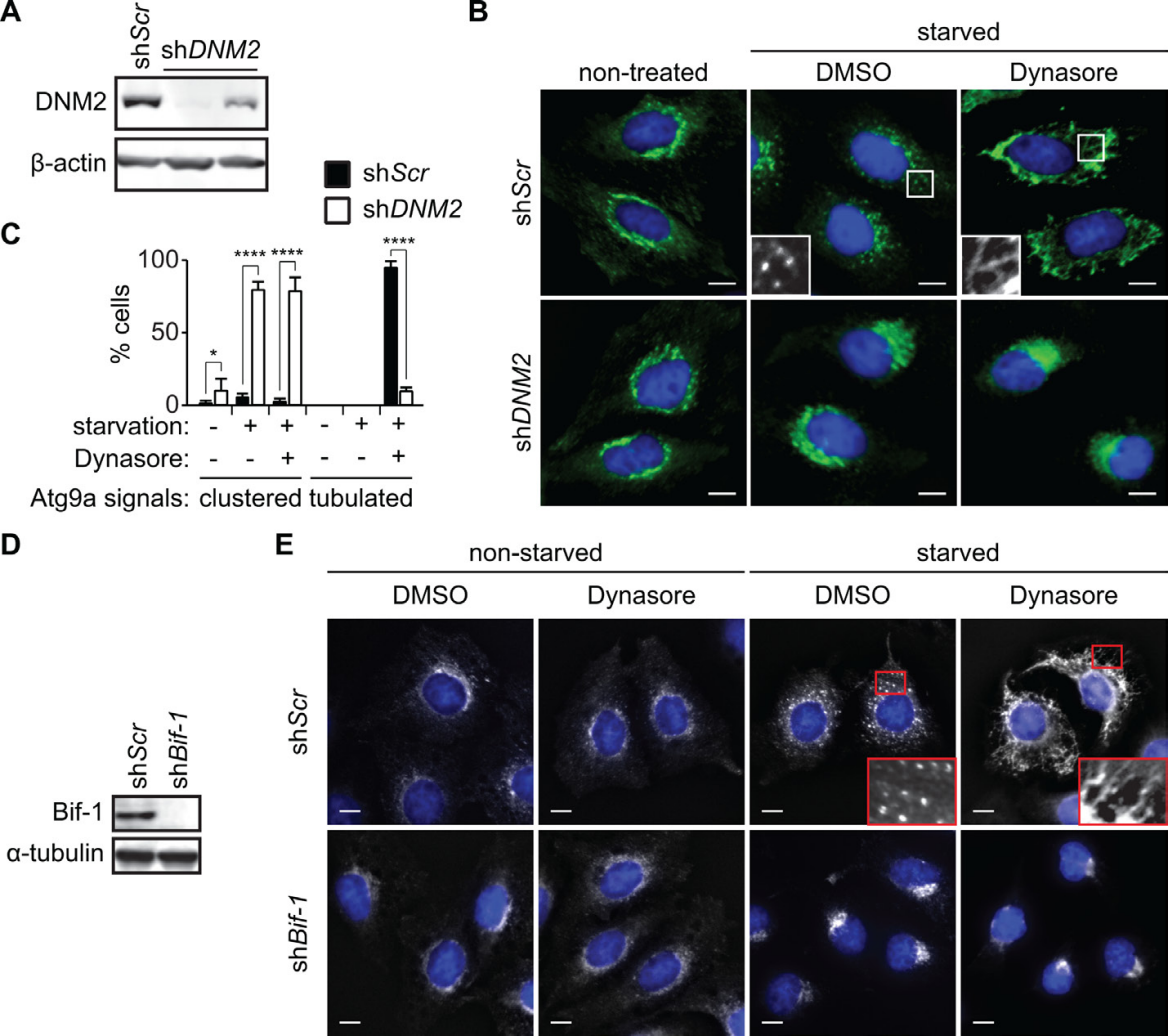
DNM2 is required for Atg9 vesicle generation induced upon nutrient starvation (**A-C**) HeLa/Atg9-GFP cells were transduced with control (sh*Scr*) or *DNM2* shRNA (sh*DNM2*) lentiviruses for 96 h. Immunoblotting was performed to analyze the expression of DNM2 (A). The cells were starved in serum and amino acid-free DMEM (starvation medium) in the presence of 80 μM Dynasore or control DMSO for 2 h and analyzed by deconvolution fluorescence microscopy (B). (C) The percentages of Atg9 vesicle generation-defective cells in which Atg9 signals clustered at the juxtanuclear region or formed tubular structures in B were calculated (*n* > 50 × 3). Statistical significance was determined by two-way ANOVA followed by multiple comparison. All values are mean ± SEM. Differences with controls were significant for *p < 0.05 and ****p < 0.0001. (**D, E**) Immunoblotting of *Bif-1* knockdown (sh*Bif-1*) and sh*Scr* HeLa/Atg9-GFP cells using the indicated antibodies (D). Cells were incubated in starvation or control medium in the presence or absence of 80 μM Dynasore for 2 h and analyzed by deconvolution fluorescence microscopy (E). In B and E, nuclei were stained with DAPI. Magnified images are shown as insets. Scale bars represent 10 μm.

During autophagy, Atg9-containing membranes undergo continuous budding to form Atg9 vesicles that are delivered to the autophagosome formation sites [9]. To determine if DNM2 is involved in this process, the expression of DNM2 was depleted in HeLa/S cells stably expressing Atg9a-EGFP (HeLa/Atg9-GFP). As shown in Figure 2A, *DNM2* shRNAs effectively suppressed the expression of DNM2. Consistent with previous findings [8, 9], Atg9 localized to the juxtanuclear region under nutrient replete conditions and translocated to vesicle-like structures in the peripheral cytoplasmic region upon nutrient starvation of control scrambled shRNA-expressing (sh*Scr*) cells (Figure 2B). In stark contrast, *DNM2* depletion abrogated Atg9 vesicle formation during starvation and resulted in clustering Atg9 signals at the juxtanuclear region (Figure 2B and 2C) in a similar manner to that observed upon loss of *Bif-1* [9]. Similar results were obtained using another shRNA targeting distinct regions of the DNM2 gene (Supplementary Figure S1A and S1B).

We next examined whether the DNM2-mediated membrane fission process is critical for generating Atg9 vesicles. As the GTPase activity of DNM2 is required for its function in membrane fission [12], we treated cells with the DNM GTPase inhibitor, Dynasore [14]. While Dynasore had minimal effect on the localization of Atg9 under non-starved conditions, treatment with the inhibitor under starvation conditions resulted in an extensive formation of Atg9-positive tubular structures (Atg9 tubules) throughout the cytoplasm (Figure 2B, 2C and 2E). Importantly, the Dynasore action is due to a specific inhibition of DNM2 activity as the depletion of *DNM2* suppressed Dynasore-induced Atg9 tubule formation (Figure 2B, 2C and S1C). Taken together, these results indicate that the large GTPase activity of DNM2 is required for membrane fission to generate Atg9-containing vesicles during autophagy.

### DNM2 cooperates with Bif-1 to induce the fission of Atg9-containing membranes

**Figure 3:**
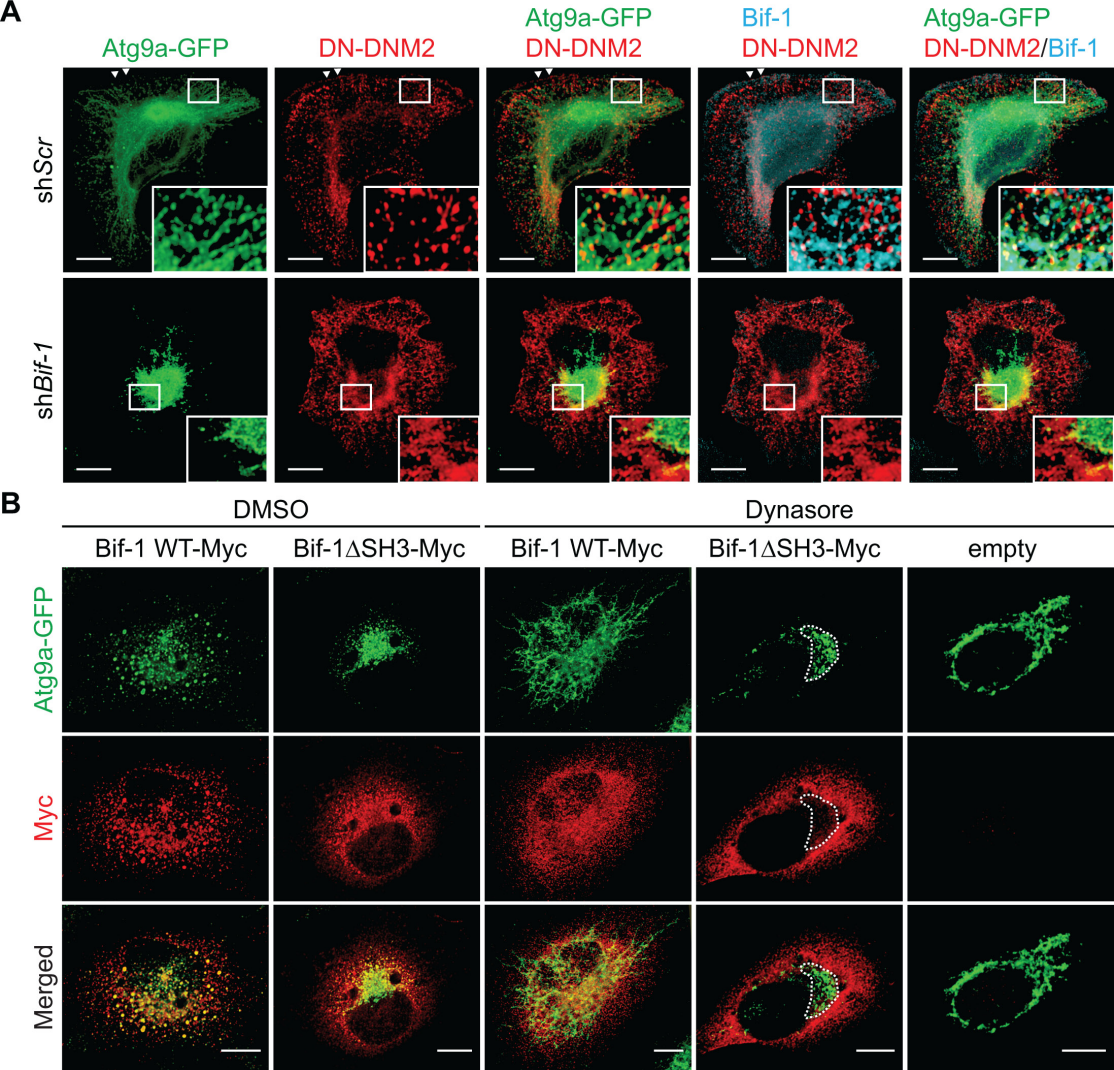
Bif-1 is important for the recruitment of DNM2 to the Atg9-containing membranes (**A**) HeLa/Atg9-GFP cells were nucleofected with *Myc-DNM2 K44E* (*DN-DNM2*) for 24 h, co-stained for Myc and Bif-1, and analyzed by confocal microscopy. Magnified images are shown as insets. Arrowheads represent DN-DNM2- and Bif-1-positive structures adjacent to the plasma membrane. (**B**) *Bif-1* knockdown HeLa/Atg9-GFP cells were nucleofected with *Bif-1* shRNA-resistant *Bif-1 WT-Myc*, *Bif-1 Bif-1ΔSH3*-Myc or control empty vector for 24 h, starved in the presence or absence of 80 μM Dynasore for 1.5 h, stained for Myc and analyzed by confocal microscopy. Dashed outline represents GFP-positive but Myc-negative region. Scale bars represent 10 μm.

We have previously reported that Bif-1 regulates the budding of Atg9 vesicles during autophagy [9]. To determine if Atg9 tubule formation induced by Dynasore is dependent on Bif-1, *Bif-1*-deficient HeLa/Atg9-GFP cells were starved in the presence of Dynasore. We found that *Bif-1*-deficient cells failed to undergo Atg9 tubule formation upon Dynasore treatment (Figure 2D and 2E). To further demonstrate the importance of Bif-1 in the DNM2-mediated Atg9 vesicle generation, we transfected HeLa/Atg9-GFP cells with a GTPase-defective, dominant-negative form of DNM2, DNM2 (K44E) (DN-DNM2) [15]. Consistent with Dynasore treatment, expression of DN-DNM2 induced the accumulation of Atg9 tubules in a Bif-1-dependent manner under normal culture conditions (Figure 3A), suggesting the importance of DNM2 in the regulation of Atg9 trafficking for basal autophagy. Moreover, DN-DNM2 signals were detected on Bif-1-positive Atg9 tubules (insets in Figure 3A), further supporting our hypothesis that DNM2 interacts with Bif-1 to regulate Atg9 vesicle generation. Interestingly, we found that *Bif-1* depletion decreased the co-localization of DN-DNM2 with Atg9 (insets in in Figure 3A) to suggest the importance of Bif-1-DNM2 interactions in the recruitment of DNM2 to Atg9-containing membranes.

To further determine the role of the Bif-1-DNM2 interaction on Atg9 vesicle formation, *Bif-1* knockdown cells were transiently transfected with *Bif-1* shRNA-resistant *Bif-1 WT-Myc*, *Bif-1*ΔSH3-Myc or control empty vector and starved in the presence or absence of Dynasore. Bif-1 WT localized to Atg9-positive structures and rescued the defect in starvation-induced Atg9 vesicle formation as well as Dynasore-induced Atg9 tubule formation in *Bif-1*-deficient cells (Figure 3B). In contrast, Bif-1 (ΔSH3) failed to localize to the juxtanuclear Atg9-positive compartment (dashed outline in Figure 3B) and generate Atg9 vesicles under starvation or Atg9 tubules under starvation in the presence of Dynasore. This result, taken together with the fact that the loss of DNM2 abrogates starvation-induced Atg9 vesicle generation or Dynasore-induced Atg9 tubule formation (Figure 2B and 2C) suggests that the SH3 domain-mediated interaction of Bif-1 and DNM2 is critical for promoting Bif-1 localization on the Atg9 vesicle budding sites for membrane tubulation. However, we do not exclude the possibility that other protein (s) that can interact with the Bif-1 SH3 domain may be responsible for the recruitment of Bif-1 on the Atg9 budding sites. Moreover, as *Bif-1* loss also decreased the colocalization of DN-DNM2 with Atg9 (Figure 3A), it is suggested that Bif-1 and DNM2 are interdependently recruited to Atg9-containing membranes to promote Atg9 vesicle generation.

### Atg9-positive compartments adjacent to the TGN serve as Atg9 reservoirs

**Figure 4:**
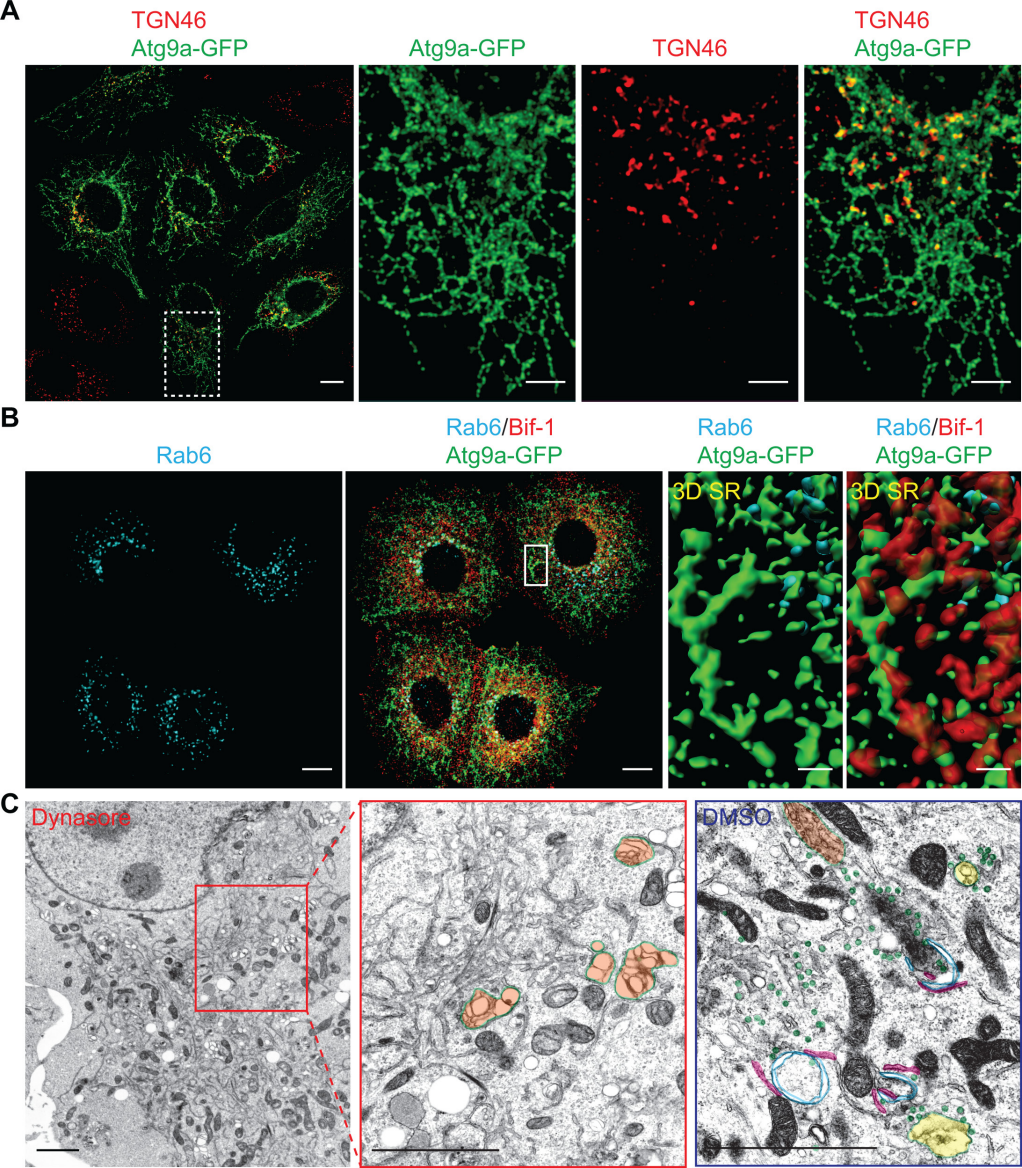
Atg9-containing membranes adjacent to the TGN tubulates upon nutrient starvation in the presence of dynasore (**A**) HeLa/Atg9-GFP cells were pre-starved for 45 min to induce Golgi fragmentation. The cells were then incubated in starvation medium containing 80 μM Dynasore for 1 h, stained for TGN46 and analyzed by confocal microscopy. Magnified images in the dash-lined boxed area are shown in the right panels. (**B**) HeLa/Atg9-GFP cells were starved in the presence of 80 μM Dynasore for 1.5 h, stained for Rab6 and Bif-1, and analyzed by confocal microscopy. 3D Surface rendered (SR) images in the boxed area are shown in the right panels. (**C**) HeLa/S cells were starved in the presence of 80 μM Dynasore or control DMSO for 1.5 h and subjected to electron microscopy. Reticular structures resembling Golgi (orange) were not surrounded with vesicles (green) as observed in control DMSO-treated starved cells but detected nearby the tubular structures. In control starved cells (DMSO), endosome-like structures (yellow) were also detected around ER (pink)-associated immature autophagosome-like structures (blue). Scale bars represent 10 μm in A and B, and 2 μm in C and the insets in A and B.

Our previous study shows that nutrient starvation induces the fragmentation of Golgi structures that are in close proximity to Atg9 signals [9]. Since DNM2 has been implicated in the post-Golgi trafficking of p75 neurotrophin receptor [15, 16], we next asked if the TGN is the source of Atg9 vesicles generated by the Bif-1-DNM2 membrane fission machinery. To this end, cells were pre-incubated in nutrient starved condition for 45 min to induce a partial Golgi fragmentation and then further starved in the presence of Dynasore. While Dynasore-induced Atg9 tubules were found in close proximity to TGN46-positive structures, TGN46 signals did not form tubular structures along with Atg9 signals (Figure 4A). Similar results were obtained by staining for Rab6, which localizes to the Golgi complex and regulates intra-Golgi and post-Golgi trafficking [17]. We found that Atg9 tubules that were positive for Bif-1 formed nearby Rab6-positive compartments (Figure 4B and Movie S1). Moreover, electron microscopy showed an accumulation of electron-dense tubular structures that are closely associated with tubular-reticular structures resembling the Golgi (orange-highlighted region) in the cytoplasmic region of Dynasore-treated cells (Figure 4C). Notably, such tubular structures were not observed in starved vehicle-control cells (Supplementary Figure S2A), but rather many vesicles resembling previously reported Atg9 vesicles (green-labeled vesicles in Figure 4C and S2B) resided in close proximity to endosome- (yellow) and Golgi (orange)-like structures as well as ER-associated immature autophagic (blue) structures (Figure 4C and S2B) [10, 18]. Immunoelectron microscopy further confirmed that Atg9 resides on both vesicle-like and immature-autophagosome-like structures in starved cells (arrowheads in Supplementary Figure S3). These results are consistent with a previous finding in yeast that Atg9 vesicles are delivered to the autophagosome formation sites and incorporated into the phagophores [19]. We also detected Atg9 signals on the juxtanuclear TGN- (orange arrows in Supplementary Figure S3) and endosome-like structures (red arrows) under control non-starved condition and a tubular-vesicular structure (green arrow) resembling to the previously proposed reservoirs [7, 10]. Taken together, these results suggest that Atg9-positive compartments that are adjacent to the TGN may serve as the Atg9 reservoirs to provide Atg9 vesicles for autophagosome formation.

### The juxtanuclear Rab11-positive endosomal compartments serve as the Atg9 reservoirs

**Figure 5:**
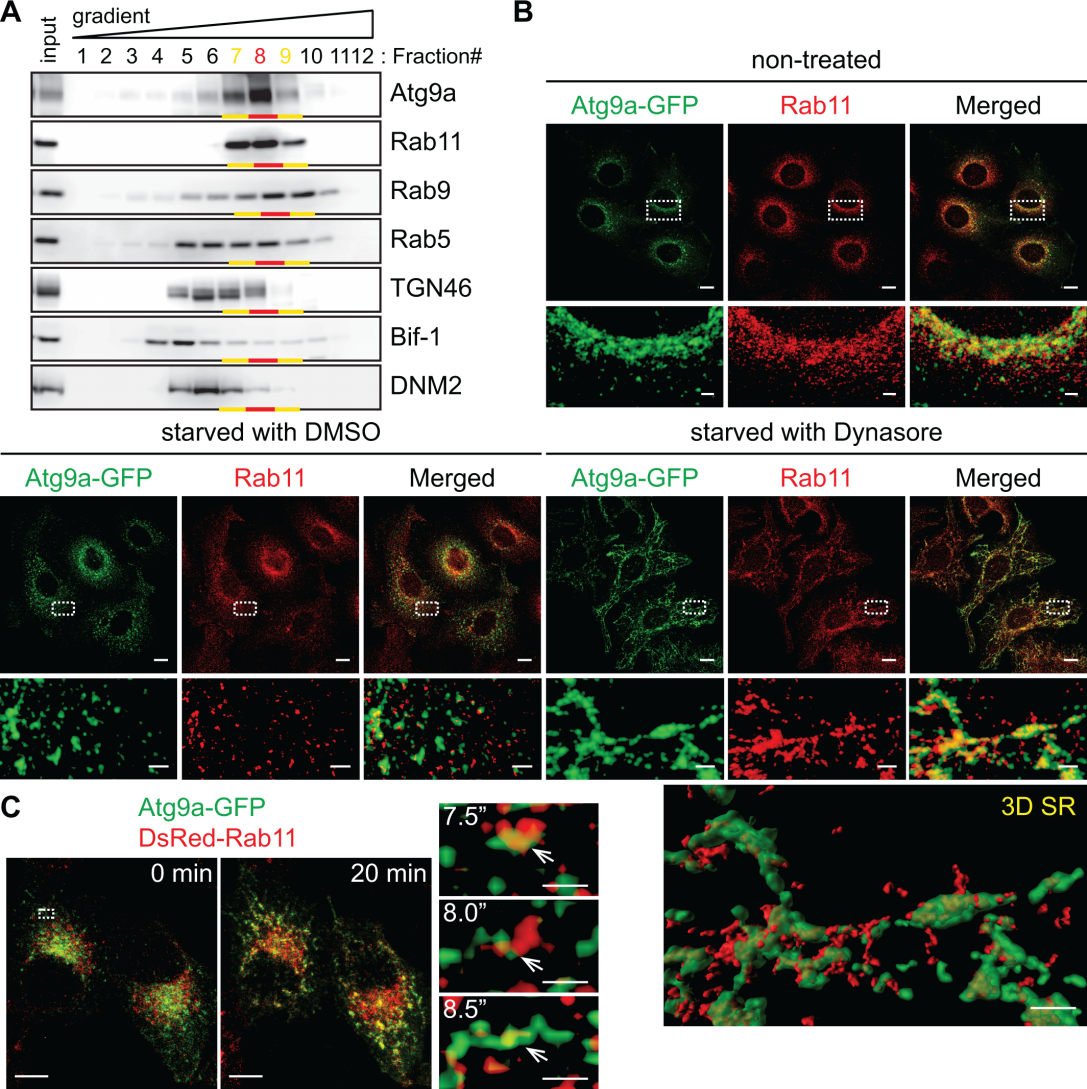
Atg9 vesicles are generated at the Rab11-positive compartments upon nutrient starvation (**A**) Post-nuclear cell homogenates prepared from HeLa/S cells were subjected to subcellular fractionation using a continuous 5∼40% OptiPrep gradient and analyzed by immunoblotting using the indicated antibodies. More than 80% of Atg9 proteins in the homogenates were fractionated in the Rab11-positive fractions (Fractions #7∼9). (**B**) HeLa/Atg9-GFP cells were incubated in complete medium or starved in the presence or absence of 80 μM Dynasore for 1.5 h, stained for Rab11 and analyzed by confocal microscopy. Magnified images in the boxed areas are shown in the bottom. 3D SR, a 3D surface rendered image of the boxed area. (**C**) HeLa/Atg9-GFP cells were nucleofected with DsRed-Rab11 for 24 h. Cells were incubated in starvation medium for 45 min and then Dynasore (80 μM) was added in the medium. 3D time-lapse images were captured at 30-sec intervals beginning 10 min after the addition of Dynasore. 3D projection images at 0 and 20 min time points are shown on the left. Magnified images in the boxed area at the indicated time points are shown in the right. Arrows indicate an Atg9 and Rab11-positive compartment that underwent tubulation upon treatment. Scale bars represent 10 μm, and 2 μm in the magnified images.

We next performed subcellular fractionation to characterize the Atg9-enriched structures that are adjacent to the TGN. It has been shown that Atg9 can localize to the early, late and recycling endosomes [8, 9, 11, 20, 21]. We found that nearly 85% of Atg9 signals were detected in the fractions #7∼9, which were indeed positive for the endosomal markers Rab5 (early endosome), Rab9 (late endosome) and Rab11 (recycling endosome) (Figure 5A). Since Atg9a and Rab11 showed very similar migration patterns in an OptiPrep gradient, we asked if the Rab11-positive compartment is the reservoir to provide Atg9-containing vesicles during autophagy. Immunofluorescence microscopy revealed that the juxtanuclear Atg9 signals under non-starved condition were positive for Rab11 (Figure 5B). Moreover, a portion of Atg9-containing vesicles that were dispersed upon nutrient starvation remained positive for Rab11 (starved in the presence of control DMSO in Figure 5B). Notably, we observed that Atg9 tubules generated during nutrient starvation in the presence of Dynasore were also positive for Rab11 (Figure 5B and Movie S2). Time-lapse imaging further revealed the tubulation of Atg9 and Rab11-positive compartments within 10 min after the addition of Dynasore to nutrient-starved cells (7.5”∼8.5” in Figure 5C and Movie S3). Notably, while *Bif-1* depletion did not inhibit Atg9 localization to Rab11-positive compartments (Supplementary Figure S4A), the amount of DNM2 in these compartments was significantly lower in *Bif-1* knockdown cells as compared to control cells (Figure S4B). This result is consistent with the immunofluorescence microscopy data in which Bif-1 deficiency reduced the colocalization of Atg9a and DN-DNM2 (Figure 3A). Importantly, both Atg9 and Rab11 signals failed to generate tubules upon starvation and Dynasore treatment in the absence of *Bif-1* (Supplementary Figure S4C). Taken together, these results indicate that the juxtanuclear Rab11-positive endosomal compartments can serve as reservoirs for the generation of Atg9 vesicles during autophagy.

**Figure 6:**
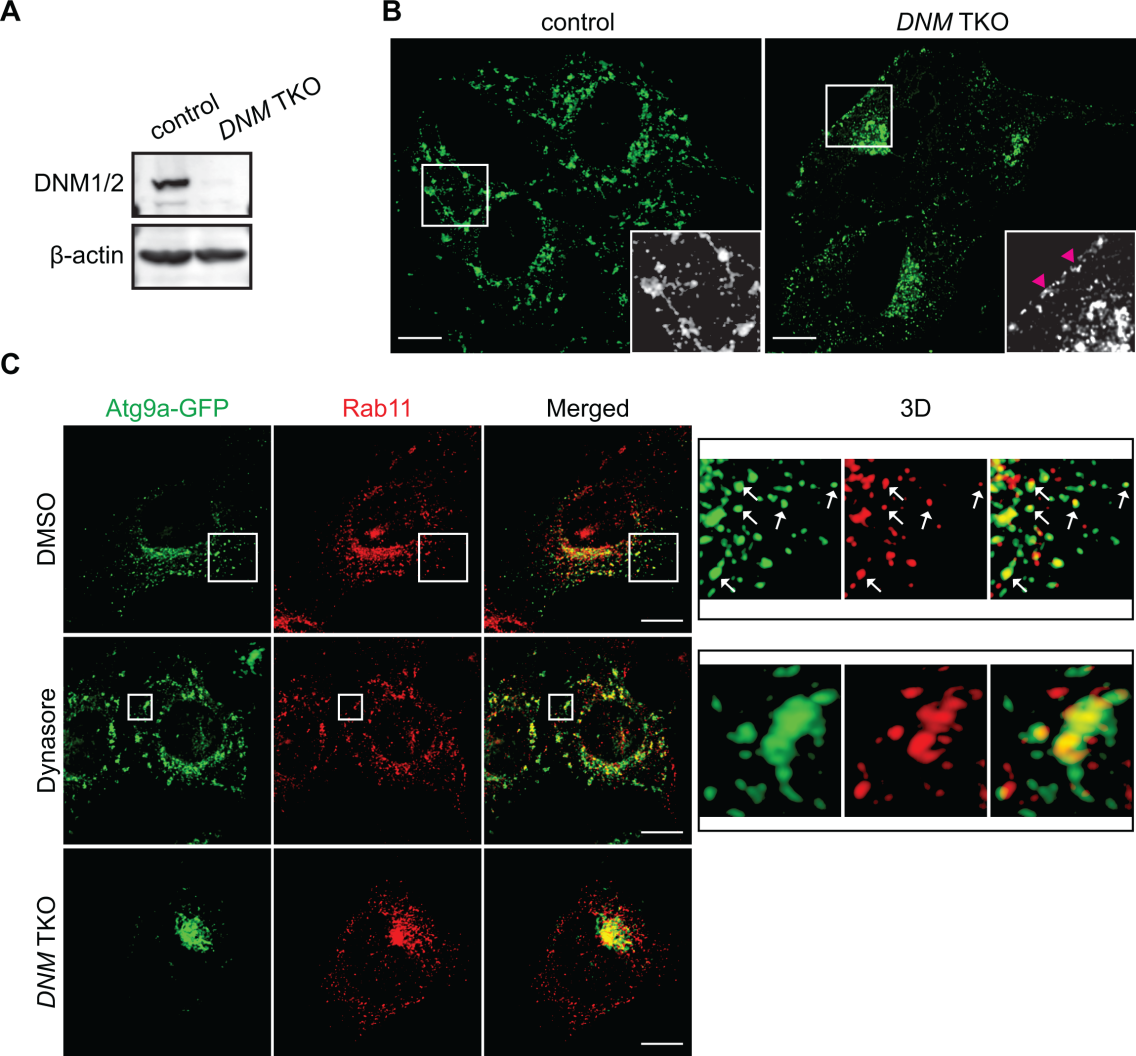
The DNM-mediated membrane fission machinery regulates Atg9 vesicle generation at the Rab11-positive compartments Tamoxifen-inducible DNM triple KO (TKO) mouse fibroblasts stably expressing Atg9a-GFP were generated as described in the Materials and Methods. (**A**) Depletion of DNMs were analyzed by immunoblotting using the indicated antibodies. (**B**) The cells were pre-starved for 45 min and then starved in the presence of 120 μM Dynasore for 1 h. The fluorescence images were analyzed by confocal microscopy. Arrowheads represent Atg9 signals adjacent to the plasma membrane. (**C**) The cells were pre-starved for 45 min, starved in the presence of 120 μM Dynasore or control DMSO for 1 h, stained for Rab11, and analyzed by confocal microscopy. 3D images of the boxed areas are shown in the right panels. Arrows indicate Atg9- and Rab11-positive vesicle-like structures. Scale bars represent 10 μm.

To further validate the importance of the DNM-mediated fission machinery in the regulation of Atg9 vesicles at the Rab11-positive compartments, we next employed an inducible *DNM* knockout system [22]. We first transduced tamoxifen-inducible *DNM* triple KO (TKO) mouse fibroblasts with lentiviruses encoding Atg9a-EGFP. The resultant cells were treated with or without tamoxifen to obtain TKO/Atg9-GFP and control/Atg9-GFP cells. Depletion of *DNMs* was determined by immunoblotting (Figure 6A). Consistent with the results obtained using HeLa/S cells, while nutrient starvation resulted in the translocation of Atg9 vesicles to the peripheral cytoplasmic region, the addition of Dynasore to nutrient-deprived control cells induced Atg9 tubule formation (Figure 6B) although the tubules appeared to be much shorter than those in HeLa/S cells (Figure 4A). Similar to that in HeLa/S cells, many of the cytoplasmic Atg9 vesicles as well as the Dynasore-induced Atg9 tubules in starved control cells were positive for Rab11 (Figure 6C). Most importantly, depletion of *DNMs* resulted in the accumulation of Atg9 signals at the juxtanuclear Rab11-positive compartments to indicate the importance of DNM for Atg9 membrane tubulation and vesicle formation during starvation. Collectively, these results demonstrate that the generation of Atg9 vesicles during autophagy occurs from Rab11-positive compartments in a DNM-dependent manner.

### Loss of *DNMs* or *Atg9a* accumulates LC3-II and suppresses p62 degradation upon starvation

**Figure 7:**
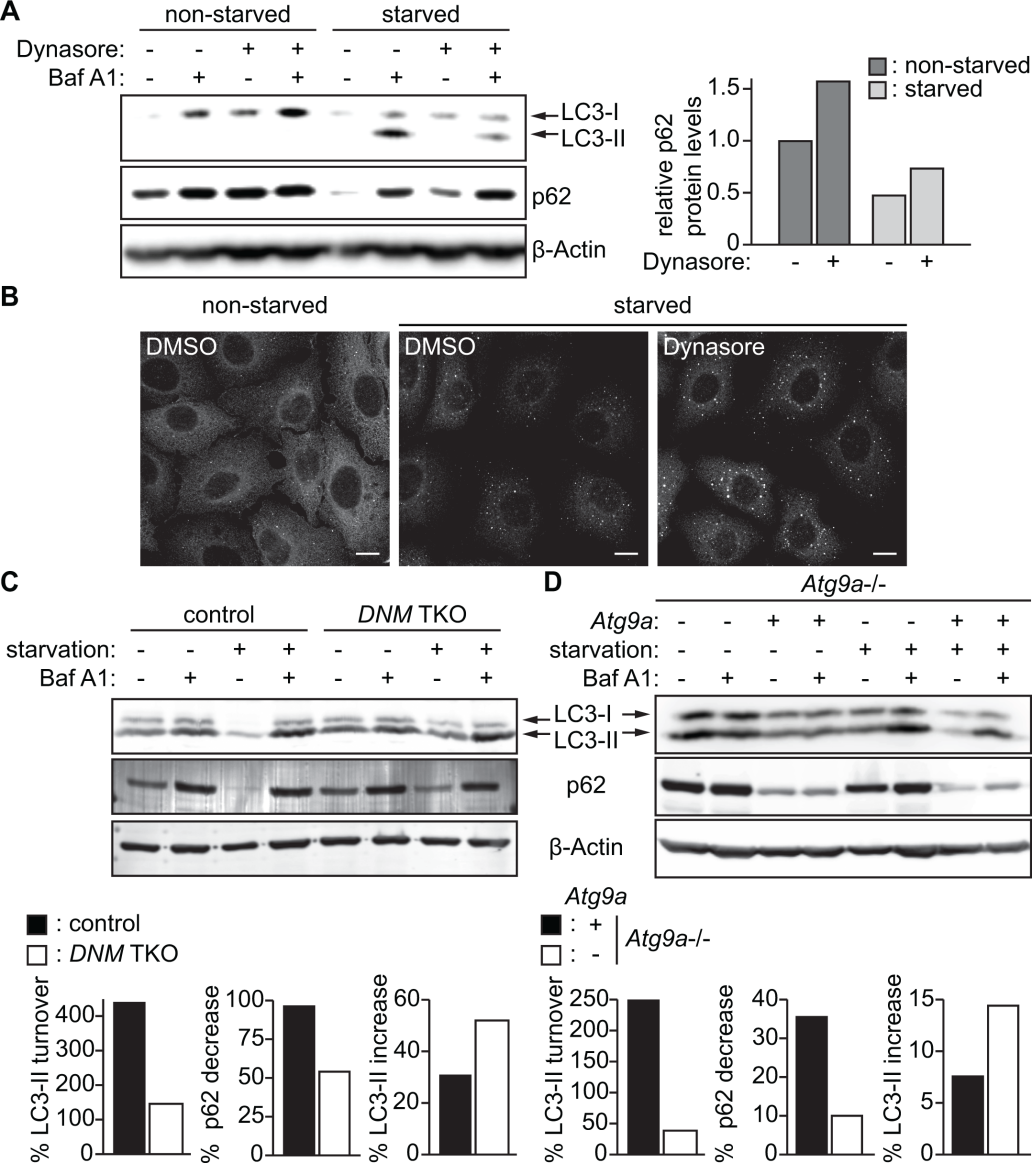
Impairment of the DNM-mediated Atg9 vesicle generation process inhibits autophagy (**A**) HeLa/S cells were incubated in starvation or control complete medium in the presence or absence of 80 μM Dynasore and 100 nM Bafilomycin A1 (BafA1) for 2 h and subjected to immunoblotting using the indicated antibodies. (**B**) HeLa/S cells were starved in the presence or absence of 80 μM Dynasore for 2 h and stained for p62. The scale bars represent 10 μm. (**C**) DNM TKO and control mouse fibroblasts or (**D**) *Atg9a*–/– mouse embryonic fibroblasts stably expressing Atg9a-PTP or their parental cells were starved in the presence or absence of 100 nM BafA1 and subjected to immunoblotting using the indicated antibodies. The starvation-induced degradation of p62, the lysosomal turnover of LC3-II and the increase of LC3-II during starvation in the presence of Baf A1 were calculated as described in the Materials and Methods.

We next explored the importance of the DNM-mediated fission machinery in autophagy by monitoring the turnover of a well-characterized autophagosomal membrane marker, LC3-II, and the degradation of an autophagic substrate, p62 [23]. During autophagy, a cytosolic form of LC3 (LC3-I) is lipidated to become LC3-II that localizes on both the outer and the inner surfaces of the phagophore; the outer membrane-bound LC3-II is delipidated by the cysteine protease Atg4 upon phagophore closure, whereas the inner membrane-bound LC3-II is degraded upon autophagosome-lysosome fusion [24–26]. p62/SQSTM1 is an autophagic receptor that recruits ubiquitinated proteins and organelles to the autophagosome and is degraded along with the cargo by lysosomal hydrolases [27, 28]. As shown in Figure 7A, inhibition of lysosomal degradation by Bafilomycin A1 (BafA1) significantly increased LC3-II in starved control HeLa/S cells. However, the increase in LC3-II was suppressed by Dynasore treatment, indicating an impairment of LC3-I lipidation. Consistently, p62 levels were increased in Dynasore treated cells under both nutrient-rich and nutrient-deprived conditions (Figure 7A and 7B). These results suggest that Dynasore has an inhibitory effect on autophagy.

We next examined whether *DNM* depletion also impairs autophagy. We found that depletion of *DNMs* in mouse fibroblasts impaired the lysosomal turnover of LC3-II during starvation (Figure 7C). Consistently, the starvation-induced degradation of p62 was suppressed in *DNM*-deficient cells. Notably, in contrast to Dynasore treatment, depletion of *DNMs* did not suppress LC3-I lipidation. This suggests that the impairment in LC3-I lipidation observed by Dynasore treatment (Figure 7A) could be due to off-target effects of Dynasore. To further determine the role of Atg9 vesicles in autophagosome formation, we examined the effect of *Atg9*a deficiency on autophagic flux. To avoid clonal variations, we restored the expression of Atg9a in *Atg9a*-deficient MEFs by transducing with lentiviruses encoding *Atg9a-PTP*. Consistent with the results obtained using *DNM*-deficient cells, the starvation-induced lysosomal turnover of LC3-II and p62 degradation, but not LC3-I lipidation, were inhibited in *Atg9a*-deficient cells. Collectively, these results highlight the critical role of DNM-mediated Atg9 vesicle generation for autophagy.

## DISCUSSION

It has recently been proposed in yeast that Atg9 is pooled at the unidentified tubular-vesicular structure, known as the Atg9 reservoir and traffics to the autophagosome formation site upon the induction of autophagy [7]. Similarly, mammalian Atg9 has been found on tubular-vesicular compartments resembling endosomes [9–11]. However, the molecular machinery responsible for generating Atg9 vesicles during autophagy remains unknown. We have previously reported that juxtanuclear Atg9-positive compartments undergo continuous budding and fission during nutrient starvation to form Atg9 vesicles in a manner that is dependent on a membrane curvature-inducing N-BAR protein, Bif-1. In this study, we identified DNM2 as a Bif-1 interacting protein that regulates membrane fission to generate Atg9 vesicles at the Rab11-positive reservoirs during autophagy.

Our data suggest the importance of the Bif-1-DNM2 interaction in the regulation of Atg9 vesicle budding and fission upon autophagy induction. The interaction of Bif-1 with DNM2 is dependent on the SH3 domain of Bif-1 and is enhanced by nutrient starvation. Moreover, Bif-1 is important for DNM2 localization on Atg9-containing membranes. We have found that DNM2 is required for nutrient starvation-induced Atg9 vesicle generation and inhibition of the DNM2 GTPase activity accumulates Atg9-positive tubular structures in a Bif-1-dependent manner and impairs autophagic flux. The observation that *Bif-1* depletion suppresses Atg9 tubule formation during DNM2 inhibition is consistent with the idea that Bif-1-induced membrane curvature drives the budding of Atg9-containing membranes to facilitate membrane fission [9]. Interestingly, while Bif-1 regulates DNM2 localization as described above, our results also suggest the importance of DNM2 in the recruitment of Bif-1 to Atg9 reservoirs to initiate vesicle budding. While deletion of the Bif-1 SH3 domain has no impact on membrane-binding activity of Bif-1 [9], Bif-1ΔSH3 fails to localize on the Atg9 reservoir or induce vesicle budding. Moreover, depletion of either *Bif-1* or *DNM2* is sufficient for inhibiting Atg9 vesicle budding upon starvation. Based on these findings, we hypothesize that the interaction of Bif-1 with DNM2 facilitates the translocation of Bif-1 at the membrane budding site of Atg9 reservoirs to initiate membrane tubulation and to promote further recruitment of DNM2 for subsequent membrane fission events that contribute to autophagic flux. To support this notion, cooperative recruitment of DNMs and N-BAR proteins has been suggested to regulate membrane fission during endocytosis [12]. However, we cannot exclude the role of additional SH3-domain binding proteins in the recruitment of Bif-1 to Atg9 reservoirs.

It has been proposed that recycling endosome-like structures may serve as the mammalian Atg9 reservoirs to promote autophagosome formation [10]. The importance of recycling endosomes for the phagophore formation has been further demonstrated by recent studies [11, 20]. In this study, we also have found that Atg9 is enriched in Rab11-positive compartments. By freezing Atg9-containing vesicle generation at the fission step using a DNM GTPase inhibitor, we provide further evidence that the Rab11-positive compartments can serve as the mammalian Atg9 reservoirs that undergo Bif-1-dependent tubulation during nutrient starvation. Furthermore, loss of *DNM2* (or all three *DNM*s) results in the accumulation of Atg9 in perinuclear Rab11-positive compartments. We have previously proposed that Bif-1 regulates Atg9-containing vesicle formation by mediating the fission of the Golgi membrane [9]. Notably, under nutrient-rich conditions, Atg9 cycles between the TGN, late endosome, and recycling endosome compartments [3]. Consistently, we have observed a close association of TGN46-positive structures with Atg9 tubules. However, our data show that the TGN membrane did not tubulate along with Atg9 upon nutrient starvation in the presence of Dynasore. While Atg9 can be delivered to Rab11-positive compartments independent of Bif-1 expression, our results suggest that Bif-1 and DNM2 cooperatively induce Atg9-containing vesicle formation from the endosomal compartments rather than the TGN during nutrient starvation. It is worth noting that, while microscopic analyses have shown colocalization of Atg9 signals with Golgi makers [8, 9] as well as Rab11 in the juxtanuclear region under basal conditions, subcellular fractionation revealed that the majority of Atg9 co-fractionates with Rab11 but not TGN46. These findings suggest that the majority of Atg9 may constitutively traffic out of the TGN to juxtanuclear Rab11-positive reservoirs for the starvation-induced generation of Atg9 vesicles in a Bif-1 dependent manner.

While our present data demonstrate the importance of the DNM-mediated membrane fission machinery for the regulation of Atg9 vesicles at the Rab11-positive reservoirs, DNM2 has also been proposed to regulate Atg9 vesicle generation at the plasma membrane [11, 21]. In these studies, they found that inhibition of DNM2 by Dynasore or DN-DNM2 results in the dispersion of Atg9 signals from the juxtanuclear region to the plasma membrane. We also observed that a small portion of Atg9 signals are accumulated at the plasma membrane region upon Dynasore treatment in mouse fibroblasts (arrowheads in Figure 6B); however, such redistribution of Atg9 signals was hardly observed by the loss of *DNM2* (or even all three *DNMs*) under both nutrient-rich and nutrient-starved conditions in the absence of Dynasore. Notably, dynamin GTPase inhibitors including Dynasore have been shown to destabilize F-actin, disrupts lipid raft organization and inhibit membrane ruffling in a dynamin-independent manner [22, 29]. Therefore, the discrepancy between our results and previous reports [11, 21] could be due to the dynamin-independent action of dynamin GTPase inhibitors. Along with this line, while we have observed that Dynasore treatment suppresses LC3-I lipidation upon starvation, *DNM* or *Atg9a* depletion accumulates, rather than decreases, the levels of LC3-II. This result suggests that impaired LC3 lipidation in the presence of Dynasore may be an off-target effect of the inhibitor. Nonetheless, both Dynasore treatment as well as DNM or Atg9a depletion impair the degradation of autophagic substrate p62 under basal and starvation conditions to highlight the importance of this process for autophagic flux. Moreover, the observation that loss of *DNMs* or *Atg9a* increases the levels of LC3-II upon starvation in the presence of BafA1 suggests that Atg9 vesicles may promote a lysosome-independent LC3-II turnover (e.g. the delipidation of LC3-II on the growing phagophores) to complete autophagosome formation. Interestingly, it has been reported that Atg9 colocalizes with Atg2 and Atg18 at the growing edge of the phagophore, which have been implicated in autophagosome sealing [30]. Future studies are warranted to determine if Atg9 vesicles produced by the Bif-1-DNM membrane fission machinery contribute to the sealing of the phagophore.

## MATERIALS AND METHODS

### Reagents

Antibodies were obtained from the following sources: mouse monoclonal anti-β-Actin (Sigma-Aldrich, A5441), rabbit monoclonal anti-Atg9A (GeneTex, GTX62510), mouse monoclonal anti-Bif-1 (Imgenex, IMG-265A), goat polyclonal anti-Dynamin II (Santa Cruz, sc-6400), rabbit polyclonal anti-Dynamin (Santa Cruz, sc-11362), mouse monoclonal anti-Flag (Sigma-Aldrich, F1804), rabbit polyclonal anti-Flag (Sigma-Aldrich, F7425), goat polyclonal anti-c-Myc (Santa Cruz, sc-789-G), rabbit monoclonal anti-Myc-Tag (Cell Signaling, 2278), rabbit monoclonal anti-Rab5 (Cell Signaling, 3547), mouse monoclonal anti-Rab5 (Santa Cruz, sc-46692), rabbit monoclonal anti-Rab6 (Cell Signaling, 9625), rabbit monoclo- nal anti-Rab9 (Cell Signaling, 5118), rabbit monoclonal anti-Rab11 (Cell Signaling, 5589), rabbit polyclonal anti-TGN46 (Novus Biologicals, NBP1-49643), mouse monoclonal anti-α-Tubulin (Sigma-Aldrich, T5168), goat anti-mouse IgG conjugated with Alexa Fluor 405 (Invitrogen, A31553) or 568 (Invitrogen, A11031), and goat anti-rabbit IgG conjugated with Alexa Fluor 405 (Invitrogen, A31556) or 568 (Invitrogen, A11011). The Atg9A-GFP cDNA [9] was subcloned into the Nhe I-Not I site of pCDH1-MCS1-EF1-puro lentiviral expression vector (System Bioscience, CD510A-1). Lentiviral shRNA vectors targeting human DNM2 (sh*DNM2*, TRCN0000006650, 5′- ATGTAAGTGGTGACGATTCGC-3′; sh*DNM2 #2*, TRC N0000006649, 5′-TTCGTGTTGATGTAGGACTGC-3′) were obtained from Dharmacon/Open Biosystems. Control scrambled shRNA (1864) [31] and DsRed-Rab11 (12679) [32] was obtained from Addgene. The Myc-DNM2 (K44E) expression plasmid was kindly provided by Dr. Rodriguez-Boulan (Cornell University). The Bif-1 shRNA-resistant Bif-1 WT and Bif-1 Bif-1ΔSH3 cDNA were subcloned into pEF6/Myc-His A vector as described previously [9]. Cell Line Nucleofector Kit R was obtained from Lonza (VCA-1001). Dynasore was obtained from Enzo Life Science (ALX-270-502) and dissolved in DMSO before usage. Amino acid-free Dulbecco's modified Eagle's medium (DMEM) was custom-ordered from Invitrogen. HeLa cells stably expressing Bif-1 shRNA were generated as previously described [33]. Immortalized tamoxifen-inducible conditional *DNM* triple knockout (TKO) murine fibroblasts [22] and *Atg9*a–/– mouse embryonic fibroblasts [34] were generously provided by Dr. De Camilli (Yale University School of Medicine) and Drs. Saitoh and Akira (Osaka University), respectively.

### Cell culture and lentiviral transduction

HeLa/S cells, 293T cells, mouse fibroblasts and mouse embryonic fibroblasts were cultured in DMEM supplemented with 10% fetal bovine serum and 1× Antibiotic Antimycotic Solution (Corning, 30–004-CI). Nucleofection was performed according to the manufacture's protocol. Lentivirus-mediated gene transduction and silencing were performed as previously described [35, 36]. DNM TKO cells were generated as described previously [22]. Briefly, cells were treated with 3 μM 4-hydroxytamoxifen for 48 h and cultured for additional 3∼5 days before the usage.

### Tandem affinity purification and co-immunoprecipitation

The human Bif-1 cDNA was subcloned into the EcoRI-Xho I site of pcDNA3.4-N-R1 TAP vector [37] and transfected into 293T cells. Twenty-four hours after transfection, cells were harvested and lysed in TAP lysis buffer (50 mM Tris-HCl, pH 8.0, 150 mM NaCl, 1% Triton X-100, 2 mM EGTA, 10 mM NaF, 1 mM Na_3_VO_4_, and protease inhibitors). Cell lysates were then subjected to purification and putative Bif-1-interacting proteins were identified by mass spectrometry as described previously [38]. For co-immunoprecipitation analyses, total cell lysates were prepared either in native IP lysis buffer (0.1 M 4-Morpholineethanesulfonic acid, 1% digitonin, 0.5 mM EGTA, 1 mM Mg (CH_3_COO)_2_, pH = 6.5; for Figure 1A) or 1% Triton-X100 lysis buffer (30 mM Tris-HCl, pH 7.5, 150 mM NaCl, 10% glycerol, 1% Triton X-100 and protease inhibitor cocktail; for Figure 1B) and subjected to immunoprecipitation using anti-Bif-1 or anti-Flag monoclonal antibodies. The resulting immunocomplexes were washed three times with lysis buffer and subjected to immunoblotting with the indicated antibodies.

### Immunofluorescence, time-lapse and electron microscopy

For fluorescence and immunofluorescence microscopy, HeLa cells were grown overnight on Lab-TekII Chamber Slide (Thermo Scientific, 154917). After each treatment, cells were fixed in paraformaldehyde-PBS, permeabilized with 50 μg/ml digitonin for 10 min, blocked with 10% normal goat serum for 1 h, incubated with the primary and the secondary antibodies and mounted with ProLong Gold Antifade Mountant (Thermo Scientific, P10144 or P36941 (with DAPI)). For 3D time-lapse live cell imaging, cells were seeded on glass-bottom microwell dishes (MatTek, p35GCOL-0–14-C) after nucleofection, cultured for 24 h and pre-starved for 45 min followed by the addition of Dynasore (80 μM) to the medium. 3D images were taken at 30-sec intervals using a Leica AOBS SP8 laser-scanning confocal microscope with a heated CO_2_ chamber. Fluorescent images were obtained using an OLYMPUS IX81 deconvolution microscope (63× oil-immersion lens) or a Leica AOBS SP8 laser-scanning confocal microscope (63× oil-immersion lens), deconvolved using SlideBook software (Intelligent Imaging Innovations) or Huygens deconvolution software (Scientific Volume Imaging) and analyzed using SlideBook software or and Imaris software (Bitplane). Electron microscopy was performed as described previously [36].

### Subcellular fractionation

Subcellular fractionation was performed as described previously [9]. Briefly, cell homogenates were prepared in homogenization buffer (0.25 M sucrose, 140 mM NaCl, 1 mM EDTA, 20 mM Tris-HCl, pH 8.0) containing protease and phosphatase inhibitor cocktails, passed through a 27-guage syringe needle and then centrifuged at 500 × *g* at 4°C in order to obtain post-nuclear cell homogenates. The resultant post-nuclear cell homogenates (0.8 ml) were loaded on 6.4 ml of a 5–40% continuous OptiPrep (SIGMA, D1556) gradient and centrifuged at 36,000 rpm for 20 h at 4°C using a SW-41 swing rotor. Twelve fractions were collected from the top of the gradient and analyzed by immunoblotting with the indicated antibodies.

### Autophagic assays

Tissue lysates were prepared in radio-immunoprecipitation assay buffer (150 mM NaCl, 10 mM Tris-HCl, pH 7.4, 0.1% SDS, 1% Triton X-100, 1% Deoxycholate, 5 mM EDTA, pH 8.0) containing protease and phosphatase inhibitors and subjected to immunoblotting The intensity of p62 and LC3-II were quantified by densitometry using Image Studio 5.0 software (LI-COR Biosciences) and normalized to β-actin. The percentages of (1) starvation-induced p62 decrease, (2) the lysosomal turnover of LC3-II under starvation and (3) the increase of LC3-II during starvation in the presence of BafA1 were calculated as follows: (1) (non-starved – starved)/non-starved × 100; (2) (starved with BafA1 – starved)/starved with BafA1 × 100; (3) (starved with BafA1 – non-starved with BafA1)/starved with BafA1 × 100.

## SUPPLEMENTARY MATERIALS FIGURES AND MOVIES








