# Supplementary material for: The Bif-1-Dynamin 2 membrane fission machinery regulates Atg9-containing vesicle generation at the Rab11-positive reservoirs
Source: Oncotarget. 2016 Mar 10;7(15):20855–68. doi: 10.18632/oncotarget.8028 (PMC4991497; doi:10.18632/oncotarget.8028)
Supplement: Supplementary file 1 [file oncotarget-07-20855-s001.pdf]

The Bif-1-Dynamin 2 membrane fission machinery regulates Atg9-containing vesicle generation at the Rab11-positive reservoirs

Supplementary Materials

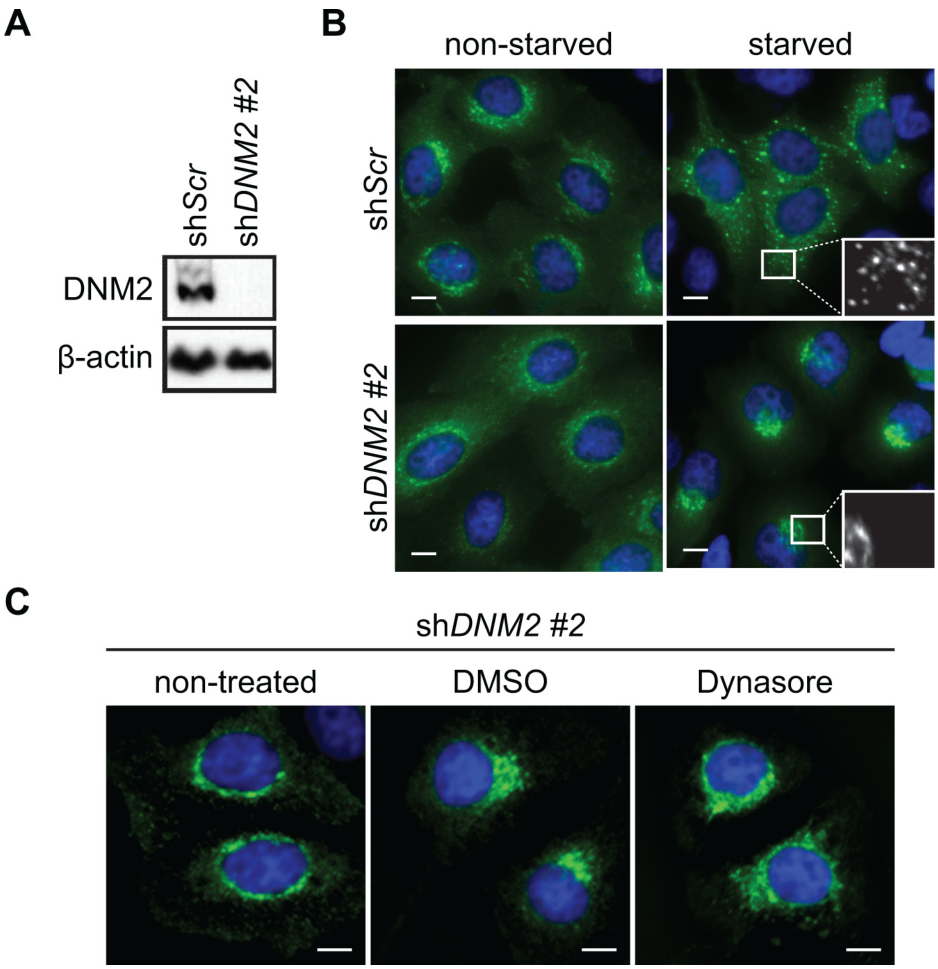

Supplementary Figure S1: DNM2 regulates the budding of Atg9 vesicles upon nutrient starvation. HeLa/Atg9-GFP cells were transduced with control (sh*Scr*) or sh*DNM2* #2 lentiviruses for 96 h. (**A**) Immunoblotting was performed to analyze the expression of DNM2. (**B**) The cells were incubated in starvation or complete medium for 1.5 h and analyzed by deconvolution fluorescence microscopy. (**C**) The cells were starved in in the presence of 80 µM Dynasore or control DMSO for 2 h and analyzed by deconvolution fluorescence microscopy. Scale bars represent 10 µm.

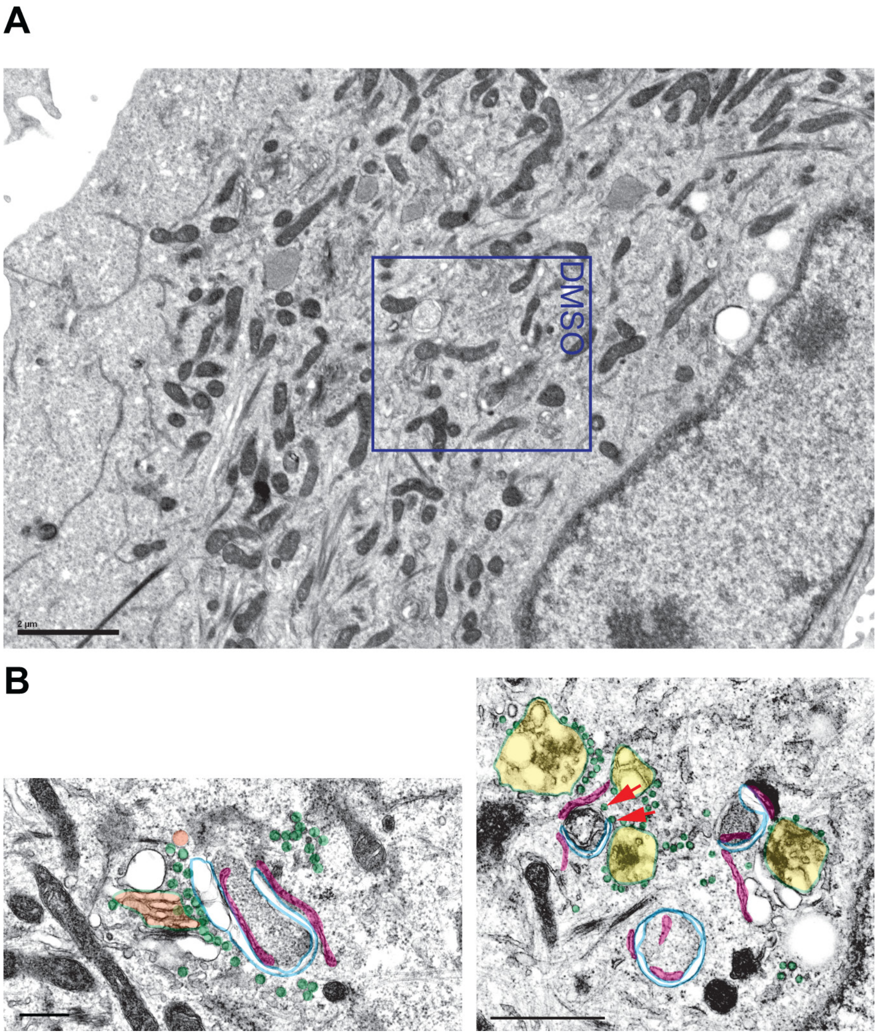

Supplementary Figure S2: Typical immature autophagosome structures observed in starved cells. HeLa cells were starved with control DMSO for 1.5 h and subjected to electron microscopy. A magnified image of the boxed area in **(A)** is shown in Figure 4. In **(B)** vesicular-reticular structures, resembling endosomes (yellow) or Golgi (orange), were observed nearby ER (pink)-associated immature autophagosome-like structures (blue). Vesicle-like structures were highlighted with green. Scale bars represent 2 µm.

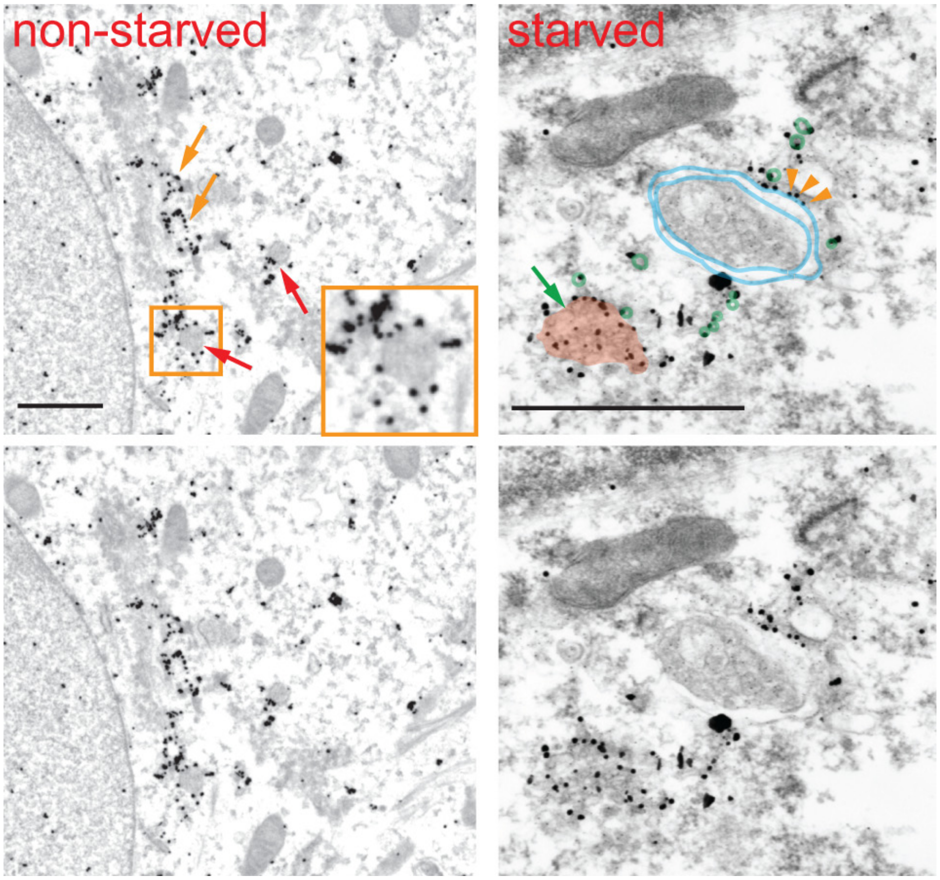

Supplementary Figure S3: Intracellular localization of Atg9a-GFP under non-starved and starved conditions. HeLa/Atg9-GFP cells were incubated in complete (non-starved) or starvation (starved) medium for 2 h and subjected to immunoelectron microscopy using anti-GFP antibodies. Scale bars represent 1 µm.

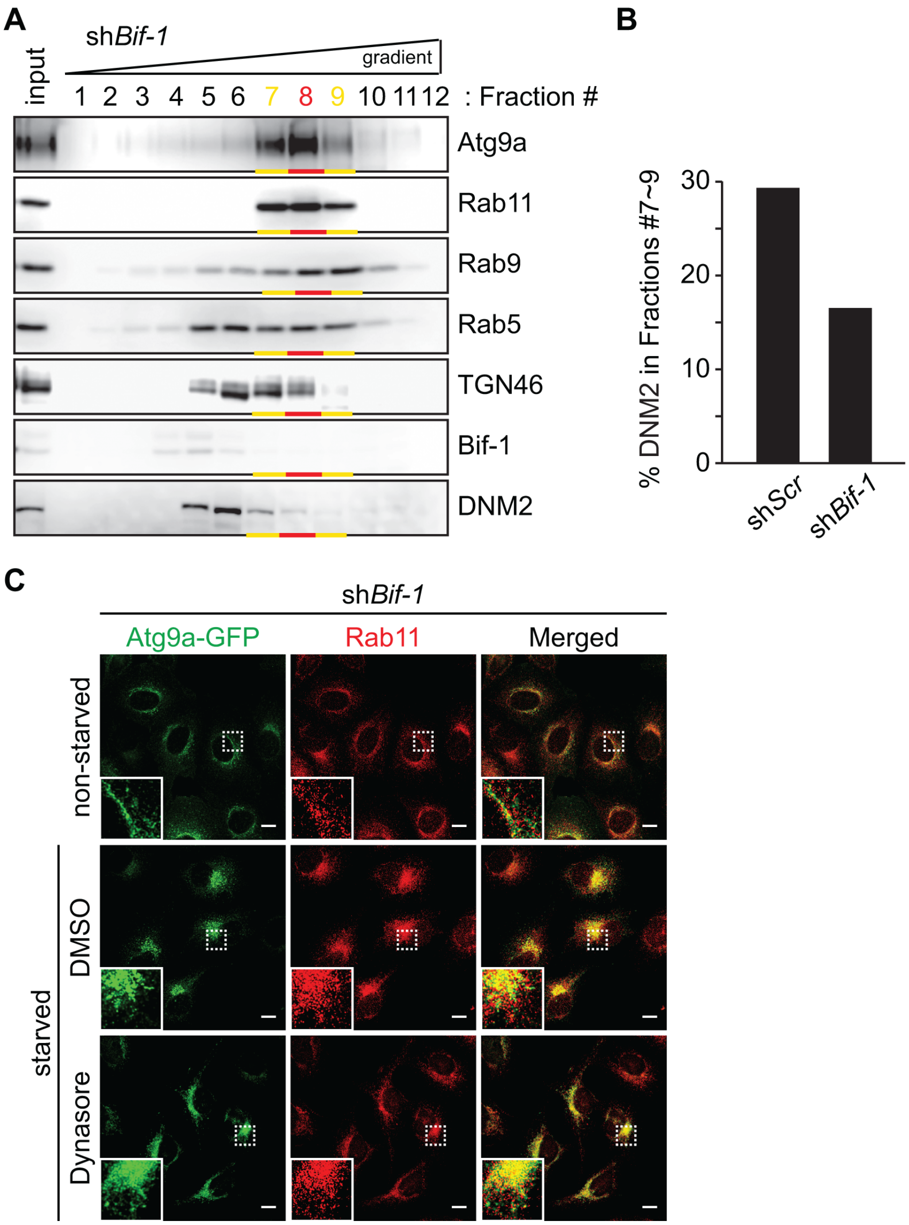

Supplementary Figure S4: Bif-1 regulates Atg9 vesicle generation at the Rab11-positive reservoir. (**A**) Post-nuclear cell homogenates prepared from Bif-1 knockdown HeLa cells were subjected to subcellular fractionation and analyzed by immunoblotting using the indicated antibodies. Similar to control HeLa cells, the majority (> 80%) of Atg9 signals were detected in Rab11-enriched fractions (Fractions #7~9). (**B**) The intensity of DNM2 in each fraction in A and Figure 5A were quantified by densitometry using Image Studio 5.0 software. The percentages of DNM2 fractionated into Fractions #7~10 to total DNM2 (Fractions #1~12) are shown. (**C**) Bif-1 knockdown HeLa/Atg9-GFP cells were incubated in complete medium or starved in the presence or absence of 80 μM Dynasore for 1.5 h, stained for Rab11 and analyzed by confocal microscopy. Magnified images were shown in the insets. Scale bars represent 10 μm.

Supplementary Movie S1: A 3D surface rendered image in the boxed area in Figure 4B.

Supplementary Movie S2: A 3D surface rendered image in the boxed area in a Dynasore-treated cell in Figure 5B.

Supplementary Movie S3: 3D time-lapse movie for Figure 5C. Green, Atg9-GFP; Red, DsRed-Rab11.
